# Ultrafast time-evolution of chiral Néel magnetic domain walls probed by circular dichroism in x-ray resonant magnetic scattering

**DOI:** 10.1038/s41467-022-28899-0

**Published:** 2022-03-17

**Authors:** Cyril Léveillé, Erick Burgos-Parra, Yanis Sassi, Fernando Ajejas, Valentin Chardonnet, Emanuele Pedersoli, Flavio Capotondi, Giovanni De Ninno, Francesco Maccherozzi, Sarnjeet Dhesi, David M. Burn, Gerrit van der Laan, Oliver S. Latcham, Andrey V. Shytov, Volodymyr V. Kruglyak, Emmanuelle Jal, Vincent Cros, Jean-Yves Chauleau, Nicolas Reyren, Michel Viret, Nicolas Jaouen

**Affiliations:** 1 Synchrotron SOLEIL, Saint-Aubin, Boite Postale 48, 91192 Gif-sur-Yvette Cedex, France; 2 Unité Mixte de Physique, CNRS, Thales, Université Paris-Saclay, 91767 Palaiseau, France; 3 Sorbonne Université, CNRS, Laboratoire Chimie Physique – Matière et Rayonnement, LCPMR, 75005 Paris, France; 4 Elettra-Sincrotrone Trieste, 34149 Basovizza, Trieste, Italy; 5 University of Nova Gorica, 5000 Nova Gorica, Slovenia; 6 Diamond Light Source, Didcot, OX11 0DE United Kingdom; 7 University of Exeter, Stocker road, Exeter, EX4 4QL United Kingdom; 8 SPEC, CEA, CNRS, Université Paris-Saclay, 91191 Gif-sur-Yvette, France

**Keywords:** Electronic properties and materials, Spintronics

## Abstract

Non-collinear spin textures in ferromagnetic ultrathin films are attracting a renewed interest fueled by possible fine engineering of several magnetic interactions, notably the interfacial Dzyaloshinskii-Moriya interaction. This allows for the stabilization of complex chiral spin textures such as chiral magnetic domain walls (DWs), spin spirals, and magnetic skyrmions among others. We report here on the behavior of chiral DWs at ultrashort timescale after optical pumping in perpendicularly magnetized asymmetric multilayers. The magnetization dynamics is probed using time-resolved circular dichroism in x-ray resonant magnetic scattering (CD-XRMS). We observe a picosecond transient reduction of the CD-XRMS, which is attributed to the spin current-induced coherent and incoherent torques within the continuously varying spin texture of the DWs. We argue that a specific demagnetization of the inner structure of the DW induces a flow of spins from the interior of the neighboring magnetic domains. We identify this time-varying change of the DW texture shortly after the laser pulse as a distortion of the homochiral Néel shape toward a transient mixed Bloch-Néel-Bloch texture along a direction transverse to the DW.

## Abstract (web summary)

There is interest in encoding of information in complex spin structures present in magnetic systems, such as domain walls. Here, Léveillé et al study the ultrafast dynamics of chiral domain walls, and show the emergence of a transient spin chiral texture at the domain wall.

## Introduction

Ultrafast demagnetization of a ferromagnet by an optical pulse was first demonstrated in 1996 in the seminal study by Beaurepaire et al.^1^, considered as the birth of the research field of femtomagnetism, i.e., the magnetism modulated (“pumped”) by femtosecond long laser pulses. While several underlying mechanisms are considered to explain these ultrafast processes, the central role of spin-dependent transport of hot electrons has been clearly evidenced^2,3^. Such phenomena were first experimentally demonstrated in spin valves, in which the demagnetization process is faster for antiparallel alignment of the magnetization in the successive magnetic layers^4^. Models based on polarized electron transport in the superdiffusive regime have been subsequently developed^5^. The optically excited hot electrons, initially ballistic, with spin-dependent lifetimes and velocities, generate non-equilibrium spin currents either within a ferromagnetic layer or in adjacent non-magnetic layers. The induced loss of angular momentum greatly participates in the ultrafast dynamical behavior of the magnetization^6^. The existence of this phenomenon has also been tested in single magnetic layers with a heterogeneous magnetization configuration, i.e., containing a large density of magnetic domains and DWs, albeit with different conclusions^6–8^. In fact, X-ray diffraction is the most powerful technique for probing the behavior of DWs at ultra-short timescales^9–11^. For example, Pfau et al.^8^ inferred the evolution of the DW width taking place in the first few ps by investigating the variations of the first-order Bragg peak of the magnetic configuration. More recently, the studies of Zuzin et al.^9^ and Hennes et al.^11^ have shown that a more precise way to extract insights about changes of DW properties is to study the position and width of higher-order diffraction peaks.

In this study, we use circular dichroism in x-ray resonant magnetic scattering (CD-XRMS) to gain access to the internal spin texture of DWs^12,13^. Thin magnetic multilayers which contain homochiral Néel DWs induced by a large interfacial Dzyaloshinskii–Moriya (DM) interaction^14,15^ are ideal systems to study chiral DW dynamics at the fs timescales. In recent studies, CD-XRMS was used^13,16–18^ to investigate the intrinsic nature of chiral DWs as well as skyrmionic systems, which is currently a topic of the utmost relevance from both fundamental and technological viewpoints^19–23^. Indeed, the amplitude of the dichroism in these experiments is not only related to the homochiral nature of the probed magnetic textures but also to the intrinsic DW configuration (Néel vs Bloch). Thus, it allows us to probe the DWs width and magnetization ratio of domain/domain wall with unprecedented sensitivity. We hence unveil the ultrafast dynamics of these chiral DWs, unambiguously showing a specific behavior compared to that of the magnetization inside the neighboring domains.

## Results

**Fig. 1:**
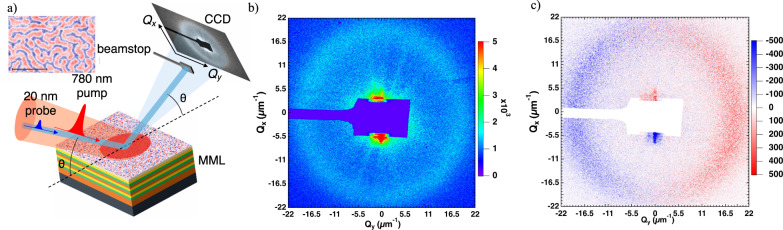
CD-XRMS experiments at Co M edge. **a** Experimental configuration with the incident beams of the IR pump and the x-ray probe. Inset: MFM phase map displaying labyrinthine domains after out-of-plane demagnetization, scale bar is 2 µm. **b** Magnetic diffraction pattern, (CL + CR). **c** Dichroic pattern (CL-CR), displaying the typical signature of clockwise Néel domain walls. The images in panels **b**, **c** have been geometrically corrected to account for the projection related to the photon incidence angle θ = 45°, and the scale corresponds to the sum of the counts (500 XFEL pulse of each polarization) for (CL + CR) (**b**) and (CL-CR) (**c**).

The studied sample is an asymmetric magnetic multilayer [Pt(3 nm)|Co(1.5 nm)|Al(1.4 nm)]_x5_ grown by sputtering deposition on a thermally oxidized Si wafer buffered by Ta(5)|Pt(5) (see Supplementary Sec. S1 for details). These multilayers have large perpendicular magnetic anisotropy and large interfacial DM interaction. At remanence, the magnetic configuration is a typical disordered labyrinthine domain configuration with a narrow distribution of domain widths (see inset of Magnetic Force Microscopy image in Fig. 1). The magnetization and anisotropy have been measured by SQUID magnetometry. The DMI amplitude has been determined by comparing the domain periodicity measured by MFM to those simulated using micromagnetic simulations with MuMax3^17,24^ (see Supplemental Material S1 for details about the magnetic preparation and the simulations) from which the DW width can be estimated to be ~20 nm. The micromagnetic simulations are also used as inputs in the empirical XRMS model with accurate values for the DW width.

The time-resolved XRMS experiments have been performed on the DiProI beamline^25^ at the FERMI free electron laser^26^. Time resolution is achieved using a standard pump-probe approach [Fig. 1a] in which the probe is a 60 fs XUV pulse at the Co *M* edge energy (photon energy ~60 eV) and the pump is a 100 fs infrared laser (IR) pulse (780 nm). The overall time resolution is therefore ~120 fs. The scattering experiments have been conducted under reflectivity condition at 45° incidence for circularly left (CL) and right (CR) x-ray polarization allowing to acquire ultrafast snapshots of diffraction diagrams (Fig. 1b) and their corresponding circular dichroism (Fig. 1c) at each delay time after the IR excitation. Note that we have used the same data analysis approach as the one described in^10^ (see SI2 for details). Noteworthy, the degree of x-ray circular polarization is between 92–95%^27^. Regarding the probe and pump energy densities, the IR fluence was set to 4.8 and 10 mJ/cm^2^ (at a repetition rate of 50 Hz) and the FEL fluence was set to 0.5 mJ/cm^2^. At the Co *M* edge, with a 45° photon incidence angle, the penetration depth is ~10 nm, therefore most of the scattered signal comes from the uppermost Co layer in the multilayered stack. Such a small penetration depth also ensures that the expected tilting of the Ewald sphere is negligible in our experiment. Finally, the experiments have been performed at the peak of the absorption resonance in order to avoid any spurious effect caused by the energy shift of the x-ray absorption spectroscopy (XAS) edge at ultrafast timescales^28,29^.

A typical diffraction pattern of the remnant magnetic configuration in our sample at negative time delays, i.e., before the laser pulse excitation, is displayed in Fig. 1b in which the diffracted intensity is the sum of the two circular x-ray polarizations (CL + CR). From the ring radius, a period of 330 ± 20 nm can be deduced (fully in agreement with the value estimated from the Fourier transformation of the MFM image). The total magnetic scattering intensity mainly comes from the alternating out-of-plane magnetic domains. The diffraction intensity shown in Fig. 1c displays circular dichroism (CL-CR), which reverses its sign on each side (along *Q*_*y*_) of the specular reflection, and reaches about 10%. Such dichroic signal is known^12^ to be a signature of the existence of a given sense of rotation in the probed noncollinear magnetic textures. As a matter of fact, the sign of the dichroism obtained here indeed reveals the stabilization of clockwise (CW) Néel DWs as we recently demonstrated in static XMRS experiments at the Co *L* edge^13^. Note that the observed features have been corroborated by similar static scattering measurements at the Co *L* edge performed at the SEXTANTS beamline at SOLEIL^30^ on the very same multilayers, for which the interpretation is now well established (see Supplementary Materials S1). We also like to point out that in what follows what is called (CL-CR) corresponds to the absolute value of the dichroism, which is azimuthally averaged over the rings’ halves (see Supplementary Materials S2).

**Fig. 2:**
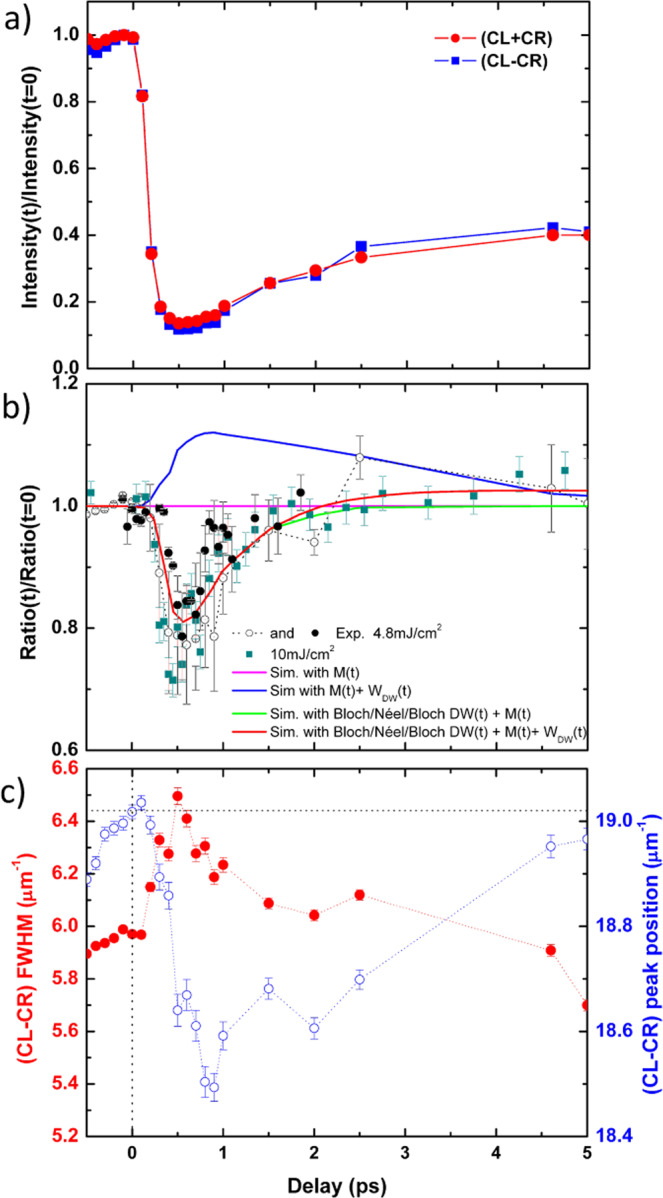
Evolution of the XRMS signal over the first 5 ps. **a** Intensity of integrated diffraction ring (CL+CR) and dichroism (CL-CR) normalized at their values at negative time delays; **b** experimental asymmetry ratio (CL-CR)/(CL+CR) normalized by its value at t < 0 in grey circles, black dots (4.8 mJ/cm^2^ IR fluence) and dark cyan square (10 mJ/cm^2^ IR fluence). The simulations for different models discussed in the main text appear as colored lines (see Supplementary Materials S3 for details). **c** Full width at half maximum (FWHM) (red dots) and the position (blue circles) in reciprocal space of the magnetic dichroic peak as a function of time.

In Fig. 2, we display the time evolution of the magnetic intensity (CL + CR) of the overall diffraction ring [red curve] and the absolute value of the dichroism (CL-CR) [blue curve]. They show a typical signature of ultrafast demagnetization in our metallic magnetic ultrathin layers, with first a quench of the magnetization reaching a minimum value after a few hundreds of fs, then followed by a log-like recovery over a few ps. In Fig. 2b, we plot the asymmetry ratio, i.e., (CL-CR)/(CL + CR) versus time. Importantly, we emphasize that this ratio should be constant in case the ultrafast dynamics occurring in the domains and in the DW would be strictly equivalent. As clearly seen in Fig. 2b (normalized by its value before the pump pulse), this is not what we observe as a 15% dip around 0.7 ps is visible. We note that the presence of such a dip in the asymmetry ratio has been reproducibly observed when repeating such XFEL experiments, as demonstrated by the overlapping series of black-filled and open circles in Fig. 2b. These different measurements display identical behavior within error bars (see Supplemental Material Section S2). An interesting behavior is that the normalized ratio remains below unity up to 2 ps.

The time evolution of the peak position and the full width at half maximum (FWHM) measuring using a Gaussian fit are displayed in Fig. 2c. A similar peak shift has already been observed in literature^9^ as well as its broadening^11^. Note however that, within our signal-to-noise ratio, we were not able to observe a potential change in the peak asymmetry as reported by Pfau et al.^8^, which could help to further discuss the link between this shift and the change of DW width.

In order to explain the observed ultrafast deviation of the asymmetry ratio, we first exclude a possible change in the scattering factors induced by hot electrons filling the *d* band. Indeed, the IR laser fluence of our experiment is much lower (~10%) than the one used to probe the change of electron occupation induced by the IR pulse using XAS^31^. Thus, our explanation is related to the fact that during the demagnetization (resp. remagnetization) process, the magnetic moments do not decrease (resp. increase) by the same amount simultaneously inside the DWs and inside the domains. In fact, in the case of a uniform decrease of magnetization after the pump, the asymmetry ratio should be constant and equal to 1, as shown by simulation using a model that is detailed in Supplemental Material S3 [magenta line in Fig. 2b]. Indeed, if one considers a DW expansion of 10% according to its width at equilibrium (20 nm), we predict an increase of the asymmetry ratio up to 1.1 as shown by the blue curve in Fig. 2b, which obviously does not correspond to the experiments. To account for an asymmetry ratio dropping below 1, we hence hypothesize a reduction of the degree of magnetic chirality i.e., a change of the ratio between the out-of-plane and the in-plane magnetization. We thus relate the ultrafast decrease of the asymmetry ratio below 1 to a different demagnetization rate between the DWs and the domains. We point out that a scenario that would correspond to a faster remagnetization of the DWs than the domains shall result in an asymmetry ratio larger than 1 (similarly to the expansion of the DW), and therefore can also not explain our result. In order to understand the experimental results, we have performed some simulations in which both the coherent evolution of the hot electron spins that induce a spin torque on the DW and the spin temperature (incoherent) variations within the DWs are included.

In fact, the understanding of the observed ultrafast DW behavior requires considering the intense flow of spin currents generated by the IR pump in the ps regime. These currents can hence efficiently transfer spin angular momentum to and from the ferromagnetic material as shown, e.g., when Pt layers absorb it and generate ps electrical pulses^32^. Angular momentum transfer and dissipation often result in both enhanced demagnetizations as well as a faster magnetization recovery. We argue that this is exactly what is happening within the noncollinear magnetic regions i.e., inside the DWs. The enhanced spin scattering within DWs has been invoked already for example for the extra contribution to the static magnetoresistance^33^ or for the induced spin-transfer torques resulting in their current-induced DW displacement. To describe these effects, ballistic models have been developed that can be appropriately adapted for the ultrafast demagnetization scenario in which superdiffusive spin currents play a central role^5^. The behavior of ballistic spin carriers can be described such as a classical spin particle perceiving a time-varying exchange field while crossing the wall^33,34^. Let us recall their salient features. First, these are band particles that are coupled by exchange to the localized spins (through the so-called *s-d* Hamiltonian). Their velocity perpendicular to the wall is related to their momentum in the *k*-space. Moreover, the localized moments are rotating in a Néel fashion within the DW. Using an appropriate parameter renormalization, the problem is equivalent to the “fast adiabatic passage” known, e.g., in NMR theory. The spin evolution is described by the Landau–Lifshitz equation:

1 $$\frac{d\vec{{{{{{\boldsymbol{\mu }}}}}}}}{{dt}}=\frac{{J}_{{ex}}S}{{{\hslash }}}\vec{{{{{{\boldsymbol{m}}}}}}}\;\times\; \vec{{{{{{\boldsymbol{\mu }}}}}}}$$

where $\vec{{{{{{\boldsymbol{\mu }}}}}}}$ is the electron spin, *J*_ex_*S* the exchange energy with the localized moment (*S*) and $\vec{{{{{{\boldsymbol{m}}}}}}}$ the direction of the time-varying exchange field seen by the ballistic electrons. Hence in the rotating frame^34^, the electronic spins are precessing around the effective field from the localized moments. Thus, they acquire a component out of the plane of rotation. The electron spin precession angle ω is proportional to the velocity *v* divided by exchange times and the DW width *2πΔ*:^33^

2 $$ < {{{{{\rm{\omega }}}}}} > =\frac{\pi {{\hslash }}v}{{J}_{{ex}}S\,2\pi \Delta }$$

For electrons at the Fermi level, this precession angle is found to be around 7 degrees for a DW width 2*π*Δ of 15 nm^34^. However, this estimation of the precessing angle can probably be quite different for hot electrons, that are the ones produced in the demagnetization process. In fact, the relevant parameter values of the hot electrons are hard to estimate. Although their velocities should not be too far from those at the Fermi level (in the 10^6^ m/s range^32^), the exchange energies in bands over 1 eV above the Fermi level can be dramatically reduced (~0.1 eV). Therefore, we anticipate that the mistracking angle could be significantly greater for a large part of the hot electrons’ distribution. All these processes shall in turn generate a torque applied on the localized moments parallel to the chiral vector: *m*_i_ × *m*_j_^35^. It is to be noticed that hot spin currents are flowing in all directions, meaning that mistracking angles can be both positive and negative. In consequence, on average, it shall result in the cancellation of the net torque acting on the DWs.

**Fig. 3:**
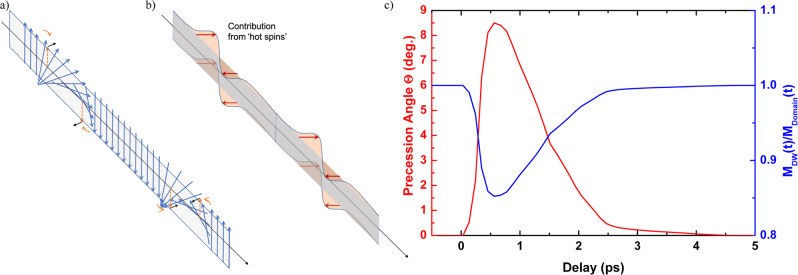
Magnetization texture modification by hot electrons. **a** Schematic representation of the torque (black arrows) imposed by the “hot spins” flowing from the domains to the DWs resulting in transient mixed Bloch/Néel/Bloch contributions. **b** Transient DW shape. **c** Precession angle (red) and DW magnetization normalized by the one from the domain (blue) used in the simulations of the asymmetry ratio shown in Fig. 2b.

To account for the observation, the overall effect of the *incoherent* precession has also to be considered. It results in an average loss of angular momentum that induces an increase in the spin relaxation processes within the DW. This effect leads to the existence after some 100 fs, of a net spin current going from the domains into the interior of the DWs. In turn, this spin current generates a torque acting on the local moments inside the DW, that is not canceled out. Importantly this torque is of opposite sign on the two sides of the DW and results in a sizeable transient tilting of the DW magnetization out of the Néel plane as shown in Fig. 3a. This phenomenon is at the origin of a new transient DW structure, made of a pure Néel type at its center together with some Bloch-type components of opposite sign on both DW sides as depicted in Fig. 3b. The presence of such a mixed Bloch/Néel/Bloch contribution in the DW internal structure is responsible for the transient reduction of the measured effective chirality as it adds two (opposite) Bloch components on both sides of the DW compared to the initially pure Néel DW structure imposed by the DM interaction. In order to estimate the amplitude of this DW distortion, we point out that unlike small current-induced electron flows at the Fermi level, spin fluxes during demagnetization are enormous as for each pulse, typically 0.5 electrons per Co atom are excited to higher bands for the used laser fluence^32^. Importantly, we note that the timescale for the onset of these induced torques is given by the exchange energy and falls in the 10-fs range, ensuring that the wall distortion does not lag from the population of hot electrons. For a spin temperature sufficiently different between domains and DWs, a quantitative estimate using the abovementioned parameters gives a precession angle of the magnetization inside the DW that is larger than 10 degrees. Moreover, the onset of this Bloch component in the DW must spill out into the domains, thus slightly increasing the effective DW width, which is a common conclusion of several recent studies^8,9,11^. In our simulations, we assume that the maximum DW width and a minimum magnetization (blue curve in Fig. 2b) take place around 1 ps. Note that this DW expansion is maximum when the quenched magnetization starts to recover (1 ps). After reaching its maximum expansion, in our simulations, we consider that the DW width then recovers its original (unpumped represented as dotted lines in Fig. 2c) size at a timescale of ~5 ps.

Using a 1D magnetization profile (described in Supplementary Material S3) and considering the experimental change of magnetization that is extracted directly from the square root of the (CL + CR) intensity, the time evolution of the asymmetry ratio can be simulated. The magnetization inside the domains is estimated from the amplitude of (CL + CR), along with a further 15% reduction of the magnetization inside the DWs to account for incoherent effects, as well as a transient Bloch–Néel–Bloch wall as shown in Fig. 3a for coherent ones. Although we cannot experimentally disentangle the two effects (coherent and incoherent) with the present data, measuring higher harmonic rings or potential satellites peaks would allow to separate these two contributions^9^. With these simulations, we find that the precession angle can reach the maximum of about 8.5 degrees after a time delay of ~0.6 ps [red curve in Fig. 3c] simultaneously with the reduction of the DW magnetization [relative to domain magnetization, see blue curve in Fig. 3c]. The resulting simulated asymmetry ratio using the described model is plotted as the green curve in Fig. 2b with an excellent agreement with the experimental measurements. Even accounting for DW expansion [red curve in Fig. 2b], the agreement can be obtained for a ~10.5 degrees tilt angle. Although the exchange-driven DW distortion is established on a very short timescale, it should last for the nanosecond timescale of the micromagnetic evolution. However, the incoherent part of the spin current shall relax at the ps timescale of the remagnetization processes, similarly to what we have measured. Interestingly, enhanced spin relaxation existing inside the DWs should speed up remagnetization, explaining that the asymmetry ratio can exceed 1, again in agreement with our experimental results.

In conclusion, we experimentally study the ultra-short timescale evolution of chiral Néel spin textures after laser-induced demagnetization in perpendicularly magnetized multilayers. Circular dichroism in x-ray resonant magnetic scattering is used to obtain information in the time domain about both the magnetic domain and domain wall configuration as well as their magnetic chirality. Beyond the evolution of the period of the magnetic domains in magnetic multilayers with large perpendicular anisotropy, we acquire new insights into the way that chirality of the noncollinear spin textures, and their long-range ordering, is evolving in the first few ps after demagnetization by a strong optical pulse. We observe that the magnetic difference CL-CR (reflecting mainly the DW properties) reduces faster than the diffracted sum signal (associated mainly with domain magnetization) in the first 2 ps after the laser pulse. To explain this unexpected change of XRMS chirality signal at such a small short timescale, we propose that transient spin current flowing from the interior of the domains inside the DWs associated with hot electrons induces an ultrafast distortion of the DW magnetization. This transient in-plane deformation of the DWs leads to a transient mixed Bloch–Néel–Bloch DW that is indeed compatible with an increase of the DWs width and a reduction of the magnetization inside the DW. These original experimental results are very well reproduced by calculations, considering a magnetization reduction of 15% with an 8.5 degrees distortion of the DW. On a longer timescale, i.e., after a few ps, we find that the DWs recover their initial chiral Néel configuration preserving the original sense of rotation (i.e., chirality) together with a recovery of their magnetization. We emphasize that our approach using dichroism in x-ray resonant scattering is applicable to any other magnetic chiral texture and should provide a better understanding of the evolution of the chirality of spin textures on the ultrafast timescale.

## Supplementary information

Supplementary Information

## Data Availability

The data that support the findings of this study are available from the corresponding author upon reasonable request.

## Code availability

The code that supports the findings of this study is available from the corresponding author upon reasonable request.
