# Supplementary material for: Ultrafast time-evolution of chiral Néel magnetic domain walls probed by circular dichroism in x-ray resonant magnetic scattering
Source: Nat Commun. 2022 Mar 17;13:1412. doi: 10.1038/s41467-022-28899-0 (PMC8931105; doi:10.1038/s41467-022-28899-0)
Supplement: Supplementary file 1 — Supplementary Information [file 41467_2022_28899_MOESM1_ESM.pdf]

Supplementary

Ultrafast time-evolution of chiral Néel magnetic domain walls probed by circular dichroism in x-ray resonant magnetic scattering.

Cyril Léveillé[1], Erick Burgos-Parra[1,2], Yanis Sassi[2], Fernando Ajejas[2], Valentin Chardonnet[3], Emanuele Pedersoli[4], Flavio Capotondi[4], Giovanni De Ninno[4,5], Francesco Maccherozzi[6], Sarnjeet Dhesi[6], David M. Burn[6], Gerrit van der Laan[6], Oliver S. Latcham[7], Andrey V. Shytov[7], Volodymyr V. Kruglyak[7], Emmanuelle Jal[3], Vincent Cros[2], Jean-Yves Chauleau[8], Nicolas Reyren[2], Michel Viret[8] and Nicolas Jaouen[1]

[1]Synchrotron SOLEIL, Saint-Aubin, Boite Postale 48, 91192 Gif-sur-Yvette Cedex, France

[2]Unité Mixte de Physique, CNRS, Thales, Université Paris-Saclay, 91767, Palaiseau, France

[3]Sorbonne Université, CNRS, Laboratoire Chimie Physique – Matière et Rayonnement, LCPMR, 75005 Paris, France

[4]Elettra-Sincrotrone Trieste, 34149 Basovizza, Trieste, Italy

[5]University of Nova Gorica, 5000 Nova Gorica, Slovenia

[6]Diamond Light Source, Didcot OX11 0DE, United Kingdom.

[7]University of Exeter, Stocker road, Exeter, EX4 4QL, United Kingdom.

[8]SPEC, CEA, CNRS, Université Paris-Saclay, 91191 Gif-sur-Yvette, France

S1. Sample growth and characterization

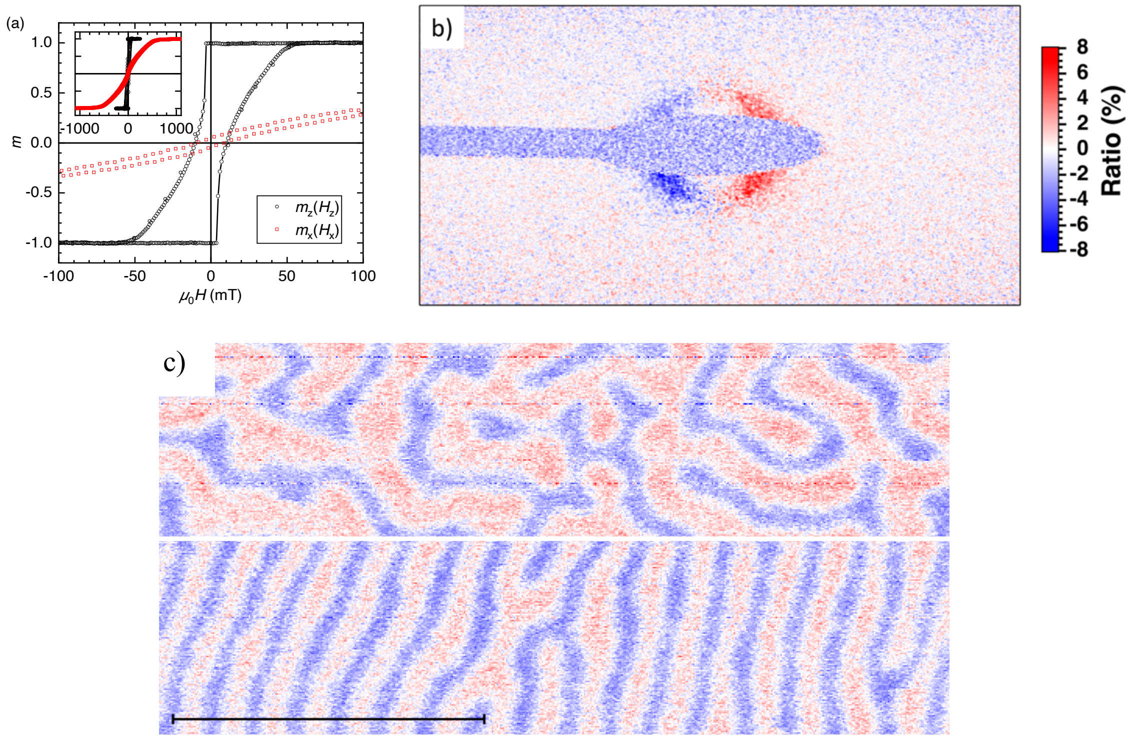

Figure S1: Characterization of the sample magnetic properties. (a) In plane (AGFM) and out-of-plane (Kerr) normalized magnetization loop. SQUID measurement on twin sample indicates that M_s = 1069 kA/m. (b) Circular dichroism in XRMS measured at Co L_3 edge (778.2 eV) at grazing incidence (theta=15.8 degree) in percent, corresponding to CW Néel DW. (c) MFM phase images after out-of-plane demagnetization (top) and in-plane demagnetization (bottom). The scale bar is 2 µm, the color scale spans 1 degree. There is a large change of period between stripe and labyrinthine domains.

The metallic multilayers are grown by magnetron sputtering at room temperature on thermally oxidized Si substrates. The sample at the center of our study is SiO_2|Ta(5)|Pt(8)|[Co(1.5)|Al(1.4)|Pt(3)]x4|Co(1.5)|Al(1.5); numbers in parentheses correspond to thickness in nm. The top Al layer is oxidized using 50 mbar of O_2 before being exposed to air. In order to maximize the photon transmission at 60 eV, but still avoiding oxidation of the Co layer, an optimal thickness of 1.5 nm Al as capping layer has been used. Reducing as much as possible the thickness of the capping is crucial for the quality of the XRMS data reported in this article. Using SQUID and alternating gradient field magnetometer (AGFM), a saturation magnetization $M_s \approx 1$ MA/m and a large out-of-plane effective anisotropy $K_{eff} \approx 0.3$ MJ/m^3 is measured. Following the approach used in Ref. [1], we checked that the sample top layer displays magnetic worm domain with a chiral clockwise (CW) Néel DW determined with XRMS measure at Co L_3 edge at the SEXTANTS beamline [2] of the SOLEIL synchrotron as displayed in Fig. S1(b).

To model the DW texture, we study the DW stripe textures (note that the period for the labyrinthine case is slightly different – we used the stripe period to limit the calculation time), we use the MuMax^3 code [3]. We used cell volumes of about $0.44 \times 0.8 \times 1.48$ nm^3 along x, y, and z directions.

The system size was ~285 × 32 × 25.1 nm^3 with periodic boundary conditions along x and y [SetPBC(32, 128, 0)]. We did not consider any electronic exchange between the magnetic layers. The magnetization is initialized with two domains, magnetization pointing along $+z$ for smaller x, and along $-z$ for larger x; the central DW is set along the [1 1 1] direction, the DW on the external side has zero width. The magnetization is minimized from this state. The set of micromagnetic parameters should be self-consistent and match several observations:

1. SQUID measurement of M_s = 1069 kA/m
2. VNA-FMR measurements indicate an effective out-of-plane anisotropy of ~296 kJ/m^3 (Sec. S4) using SQUID magnetization measurement.
3. The period of the stripes (after in-plane demagnetization) is ~290 nm as measured by MFM.
4. XRMS data indicate a clock wise DW chirality for the top layer in the disordered phase with period of about 330 nm, while the mean chirality is counter clock wise.

We calculate the uniaxial anisotropy (corresponding to the interface anisotropy) to be K_u = 1014 kJ/m^3 to match observations (1) and (2). The exchange and the effective DMI in the system are the parameters that are the more difficult to measure. Therefore, we explored the range of possible parameters that would match observation (3), i.e., the domain period p_o. This is realized by calculation of the energy areal density ε of the system for fixed parameters, but using different periods p, slightly longer or larger than the observed one. We can then calculate the numerical derivative of the energy density at the observed period p_o for a set of effective parameters for the symmetric exchange A and the DMI asymmetric exchange D (Fig. S2(a)). Finally, looking at the chirality of the top layer of the simulated multilayer DW with the XRMS period, we can check values satisfying observation (4) (Fig. S2(b)). We end up with a possible A and D parameter set: A = 10 pJ/m and D = 1.0 mJ/m^2. From other studies using Brillouin light scattering and DW propagation [1], we indeed expect $D \approx 1$ mJ/m^2 for this particular stack.

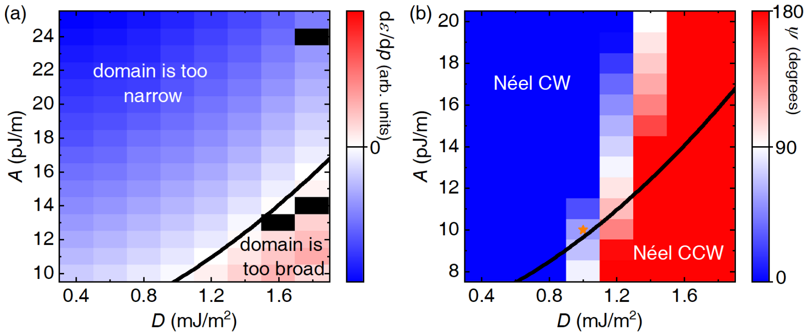

Figure S2: Determination of the exchange energies. (a) Map of the derivative of the energy density ε by the domain period p as a function of the effective DMI D and effective exchange energy A. The zero-derivative indicated by the thick black line corresponds to the set of possible parameters minimizing the energy density for the observed period p_o = 290 nm by MFM in the stripe configuration. (b) Corresponding map of the DW magnetization angle ψ of the top Co layer for the 330 nm period observed by XRMS. The curve of the possible parameters is reported from panel a. The Bloch component is determined by the initial condition of the simulation and is always around ψ = 90°. Black squares indicate discarded values. The orange star corresponds to selected parameter set for Fig. S3.

We explored in details the DW profile of the case (orange star in Fig. S2) close to the expected physical configuration, and use it to determine the width parameter of the empirical model of the XRMS analysis. In Fig. S3 are displayed the three components of the magnetization profiles. The width of the *z*-component takes the value of 18 nm. The empirical XRMS model profile shows a slight inconsistency concerning the width of the other components, taking the values 23 nm for the *x* component and 16 nm for the *y* component. The DW configuration is not purely Néel type. The Bloch "component" is achiral, meaning that it does not appear in the CD-XRMS images.

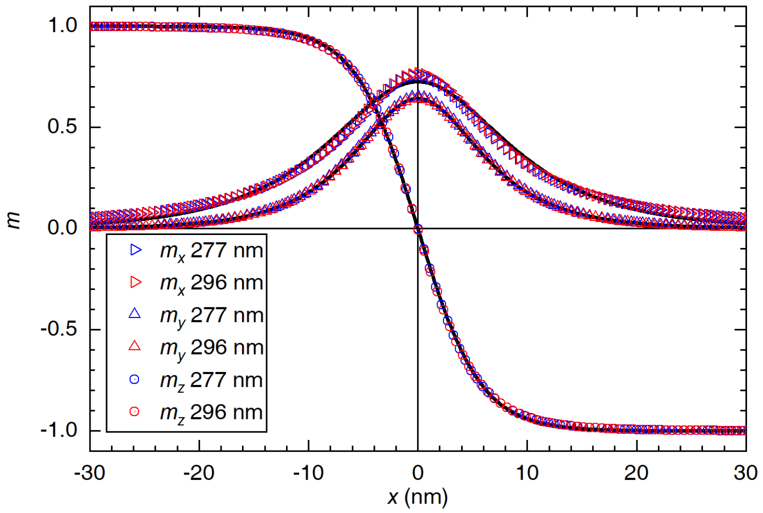

Figure S3: Magnetization profile of the minimized DWs of the top layer of the multilayer corresponding to set of parameters highlighted in Fig. S2b: A = 10 pJ/m and D = 1.0 mJ/m2. Lines are fit (in the displayed range) using the formula of Sec. S3

S2. Data Analysis

We record the scattering diagram with a PI-MTE CCD camera (2040×2048 square pixels with 13.5 µm side) from Princeton Instruments located 12 cm from the sample. For each delay between the IR laser and the XFEL pulses, we recorded 500 shots per CCD picture and stored them in an HDF5 file containing also the experiment contextual data (x-ray wavelength, FEL single shot intensity, FEL single shot spectral content, …). In order to reduce the reading time, the images have been binned 2×2 (hardware) and later renormalized (software) by the bunch intensity, a reference intensity (I_0) has been measured by recording the intensity of a photodiode placed at the entrance of the experimental end station measuring the tail of the beam for each bunch. Using these values, to avoid any artifact, we chose to remove all the x-ray bunches that differ by 20% from the average bunch intensity. Before and after each delay scan, multiple CCD background images ("darks") were performed in order to account for the CCD electrical readout and thermal noise. The averaged background image is subtracted from the measurement images.

Final images are sorted by time delay and separated by polarization, i.e., circular left and right. For each delay, the two images are resized by a geometric factor to account for the 45° incident angle. The final sum (CL + CR), difference (CL – CR), and the asymmetry ratio (CL – CR)/(CL + CR) images are then calculated. Since the FEL spot slightly moves while changing polarization, one obtains erratic points at the edge of the beamstop which affects the data analysis. Both, polar and radial masks are applied on the images to totally hide the beamstop, which consists on replacing pixel values within the range of the mask by zeros. We then extract the center of the scattering pattern as the center of the diffraction ring in the sum image. Thereafter, we perform an azimuthal averaging on the three images (we used the absolute value of CL-CR to extract the dichroic intensity). The azimuthal averaging excludes zero values outside of the beamstop radius in order to keep the full radial range of the image in the chart and to make a radial normalization by non-zero values only.

In order to remove the diffuse scattering peaking at the specular position, a large disk around the specular position is excluded from the analysis. This is particularly important for the (CL+CR) images and has a negligible influence on the dichroism (CL-CR). In order to remove the diffuse scattering background, we try several functions (gaussian, exponential or polynomial function) and we find that we obtain reliable results using either a third or fourth order polynomial function using the intervals between diffraction peaks indicated by the vertical green dotted lines as shown in Fig. S4.

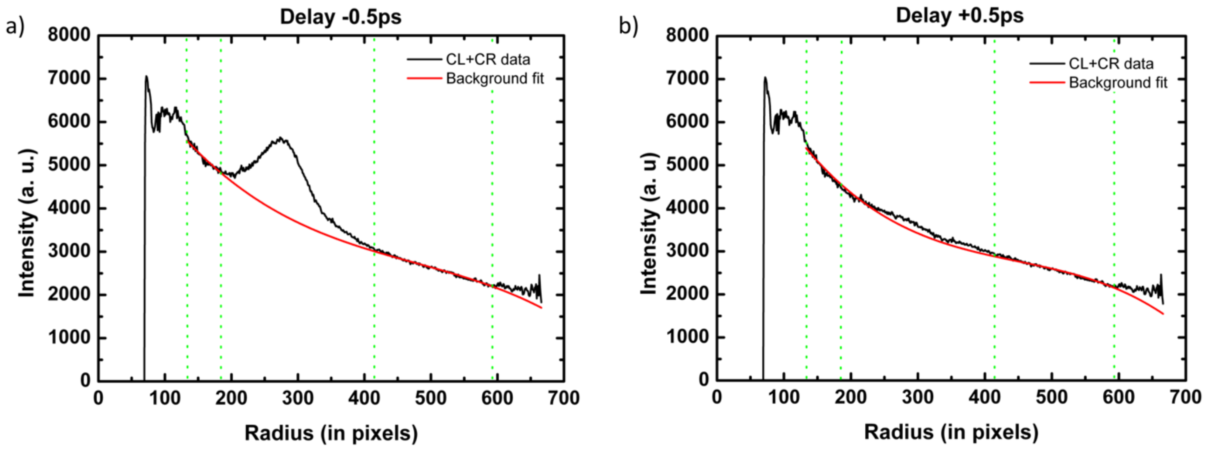

Figure S4: Typical profile of the sum image (black) for delay times of -0.5 ps (a) and 0.5 ps (b) fit with a cubic function (green line).

However, we obtain significantly different quantitative results depending the polynomial order used and in Figure 2 of the manuscript, we take the average of these extreme, and the error bars have been derived to account for this dispersion of the experimental results strongly depending on the (CL+CR) diffuse background removal.

After removing this background due to the specular spot, the diffraction ring peak is fitted. From the Gaussian fit of the dichroic signal (CL-CR) we can directly extract the values used in the main text such as the full width at half maximum (FWHM) and the position of the peaks in wave vector units (μm^{-1}).

This can also be done for the (CL+CR) intensity but with higher uncertainty. In order to illustrate the peaks shift, we display in Figure S5 the results of (CL+CR) and (CL-CR) for several delays and their corresponding Gaussian fits.

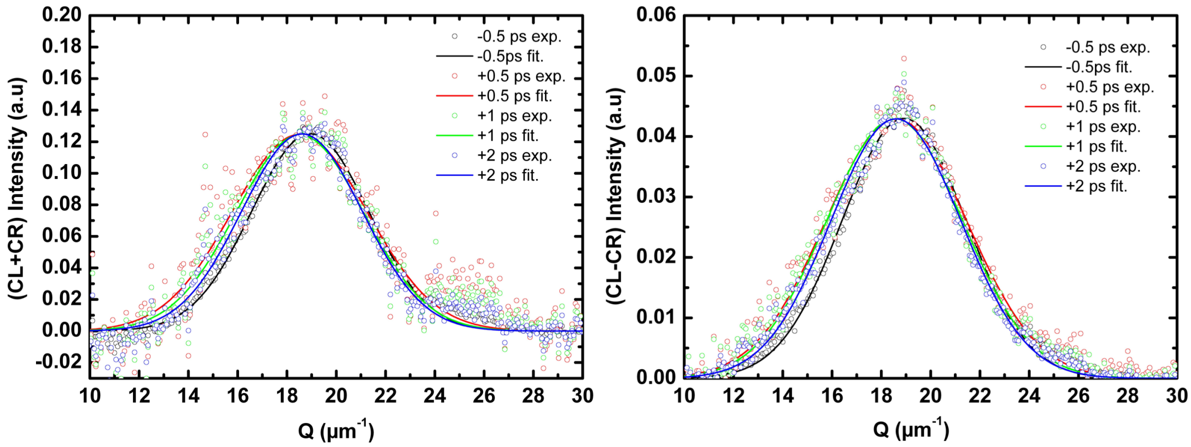

Figure S5: Fit by a gaussian function of (CL+CR) and (CL-CR) magnetic peaks before pumping (-0.5 ps), at the maximum of demagnetization (0.5 ps and 1ps) and at longer timescal (2ps). For clarity the curves and fit have been rescale to -0.5 ps one.

The orthoradial profile has also been extracted from the (CL – CR) images and displayed for several characteristic delays. Moreover, the azimuthal profile indicates that during the demagnetization time, the CW Néel type magnetic texture persists in the sample. This result is compatible with the transient mixed Bloch/Néel/Bloch DW ultrafast dynamic scenario proposed since the Bloch part is composed of opposite cancelling chiralities, thus should not appear in the diffraction pattern.

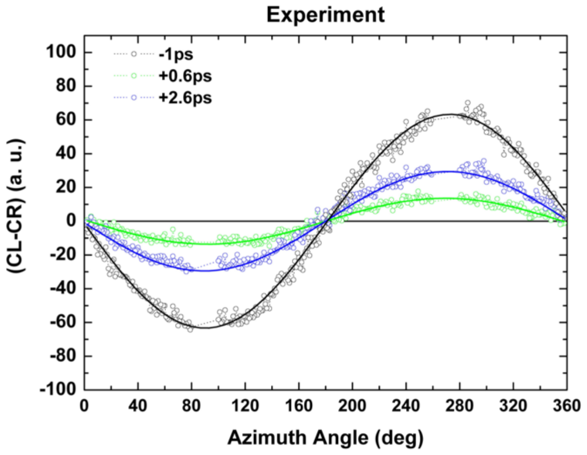

FigureS6: Orthoradial profil with beamstop mask at 90° and 270° with the origin at the top and a clockwise azimuthal angle rotation.

S3. Simulation of Asymmetry Ratio with a transient mixed Bloch/Néel/Bloch wall

The profile of a 1D mixed Bloch/Néel/Bloch is modelled as follows:

$$M_x(x,t) = \cos(\Theta(t))\, M(t) \left[\sqrt{1-\left(\tanh\left(\lambda \frac{x_1(x)}{w(t)/2}\right)\right)^2} - \sqrt{1-\left(\tanh\left(\lambda \frac{x_2(x)}{w(t)/2}\right)\right)^2} \right]; \quad (1)$$

$$M_y(x,t) = \sin(\Theta(t))\, M(t) \left[\sqrt{1-\tanh\left(\left(\lambda \frac{x_1(x)}{\frac{w(t)}{2}}\right)\right)^2} - \sqrt{1-\tanh\left(\left(\lambda \frac{x_2(x)}{\frac{w(t)}{2}}\right)\right)^2} \right]; \quad (2)$$

$$M_z(x,t) = M(t) \tanh\left(\frac{\lambda}{2} \frac{x_3}{w(t)}\right) \quad (3)$$

With $x_1(x) = \sin\left(\pi \frac{x+\frac{\lambda}{4}}{\lambda} + \frac{\pi}{4}\right)$, $x_2(x) = \sin\left(\pi \frac{x+\frac{\lambda}{4}}{\lambda} - \frac{\pi}{4}\right)$, and $x_3(x) = \sin\left(2\pi \frac{x}{\lambda}\right)$, where x is the position and λ the domain period (twice the domain size). Both are in nanometer as shown in Fig. S7(a). $\Theta(t)$ is the tilt angle arising from the torque impose by hot electrons, $w(t)$ corresponds to the time evolution of the DW width, and $M(t)$ is the temporal evolution of magnetization and is proportional to $\sqrt{CL + CR}$.

We display in Fig. S7(a) the result for the magnetization profile for $\Theta = 0°$ and $\Theta = 40°$. We calculate the scattering intensity using the resonant scattering amplitude in dipolar approximation using [5, 6, 7], without considering the charge scattering:

$$f_{EI}^{REXS} = \begin{pmatrix} 0 & \boldsymbol{k} \\ -\boldsymbol{k}' & \boldsymbol{k}' \times \boldsymbol{k} \end{pmatrix} \cdot \boldsymbol{M} \quad (4)$$

The diffracted intensity for a given incident polarization is expressed as follows [5]:

$$I = Tr\left[\tilde{f} \rho \tilde{f}^{\dagger}\right],$$

where $\tilde{f}$ and $\tilde{f}$ are the Fourier transform of the scattering amplitude f_{EI}^{REXS} and its complex conjugate, respectively, and ϱ is the density matrix of the incident x-ray beam. In the Stoke-Poincaré representation [7], the density matrix for a circularly left or right incident beam is expressed as follows:

$$\rho_{CL} = \frac{1}{2}\begin{pmatrix} 1 & -i \\ i & 1 \end{pmatrix} \text{ and } \rho_{CR} = \frac{1}{2}\begin{pmatrix} 1 & i \\ -i & 1 \end{pmatrix} \quad (5)$$

In Fig. S7(b) we represent the simulated asymmetry ratio for the several scenarii discussed in the main text. The results are normalized by the ratio simulated for a DW width of 20 nm and a 0 degree wiggling angle Θ corresponding to the static parameters issued from micromagnetic simulations (See Sec. S1 for details). The lowest value of the experimental ratio can be simulated in each model, i.e., keeping the DW magnetization equal to the domain ones, or with a reduction of the magnetization within the DW proportional to cosΘ, $\cos^2\Theta$ or to 1-sinΘ. We choose arbitrary these analytical law for illustration and for their simplicity to be inserted in the simulation but any other model could be also used.

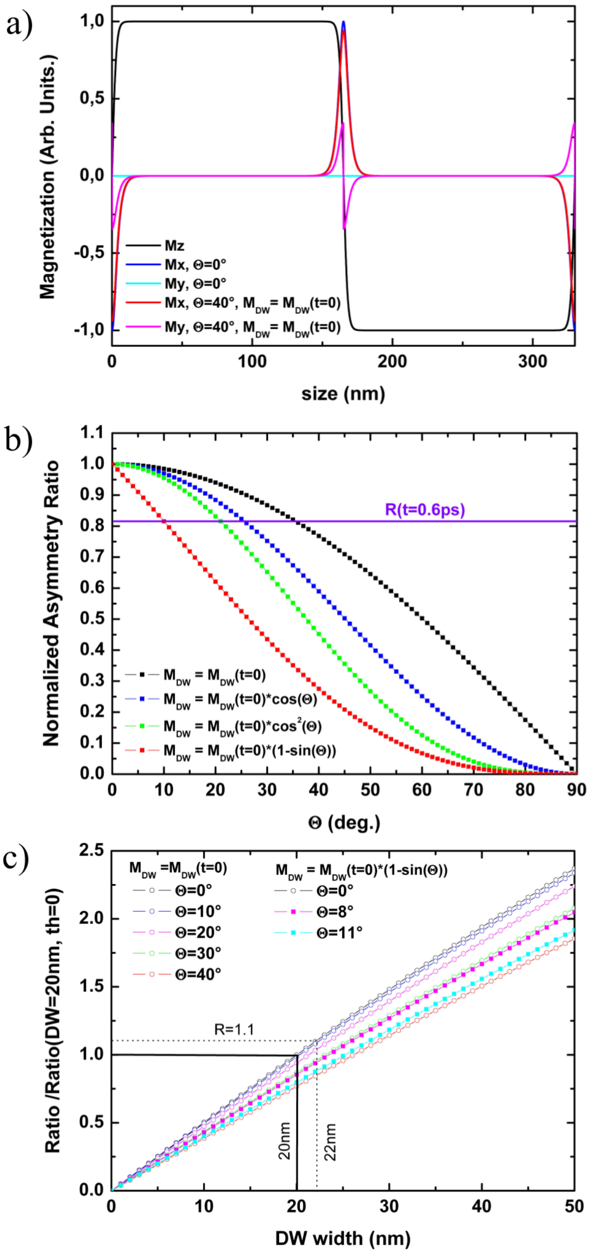

Figure S7. (a) Representation of the magnetization for Θ=0° and 40°. (b) Simulation of the asymmetry ratio (CL-CR)/(CL+CR) for different precession angle. The parameters used for the simulations are in the legend. (c) Simulation of the asymmetry ratio function of the DW size for different precessing angle Θ.

We also simulate the evolution of the magnetic asymmetry ratio as a function of the DW (Fig. S7(c)) width for a magnetization in the DW equal to the one in domain or reduced. It shows that to account for the maximum asymmetry ratio of 1.1 observed at long timescales, one has to account for a dilatation of the DW from 20 up to 22 nm. In Fig. S5(c) we represent the simulated azimuthal intensity of the asymmetry for several precession angle Θ (with a DW magnetization amplitude proportional to 1-sinΘ for different time delay according to the model discussed in the main text).

S4. VNA-FMR

Broadband vector network analyzer ferromagnetic resonance (VNA-FMR) measurements were carried out to probe the power absorbed from a radio-frequency (rf) magnetic field as a function of the rf frequency in the presence of an additional bias field. Figure S9 shows the change in rf power absorbed by the sample, which was determined from the differential of the rf transmission, S_{21}, with respect to field. The signal is weak and the results shown are obtained from an average of 10 frequency-field maps.

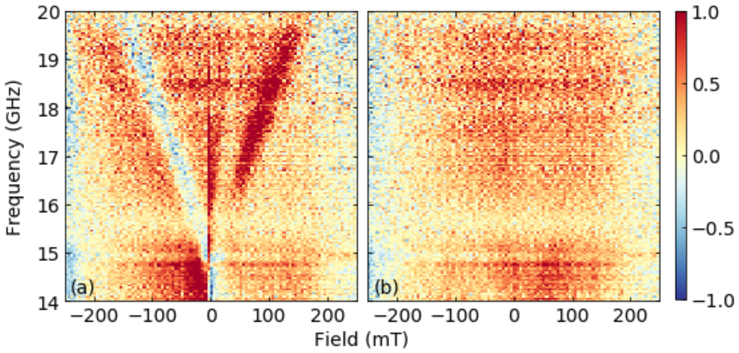

Figure S8: VNA-FMR measurement. with bias field applied (a) out-of-plane and (b) in-plane of the sample.

The figure shows VNA-FMR measurements with the bias field applied (a) out-of-plane and (b) in-plane of the sample. In both cases, the rf field is applied in-plane and perpendicular to the bias field. No features could be observed below 15 GHz (not shown). Features, which occur only for the out-of-plane field at frequencies >15 GHz, correspond to the FMR uniform precession. The value at zero field allows the effective anisotropy to be precisely estimated (see Sec. S1).

References:

[1] J.-Y. Chauleau et al., Chirality in Magnetic Multilayers Probed by the Symmetry and the Amplitude of Dichroism in X-Ray Resonant Magnetic Scattering. Phys. Rev. Lett. 120, 037202 (2018).

[2] M. Sacchi et al., The SEXTANTS beamline at SOLEIL: a new facility for elastic, inelastic and coherent scattering of soft X-rays. J. Phys. Conf. Ser. 425, 072018 (2013).

[3] A. Vansteenkiste et al. The design and verification of MuMax3. AIP Adv. 4, 107133 (2014).

[5] J. P. Hannon, et al., X-Ray Resonance Exchange Scattering. Phys. Rev. Lett. 61, 1245 (1988).

[6] J. P. Hill and D. F. McMorrow, Resonant Exchange Scattering: Polarization Dependence and Correlation Function. Acta Crystallogr. Sect. A 52, 236 (1996).

[7] G. van der Laan, Soft X-ray resonant magnetic scattering of magnetic nanostructures. C.R. Phys. 9, 570 (2008).
